# Postoperative Visual Outcome: Sling procedure with prolene sutures in children with simple congenital ptosis

**DOI:** 10.12669/pjms.38.1.4359

**Published:** 2022

**Authors:** Nida Shamim, Nausheen Hayat, Alyscia Cheema

**Affiliations:** 1 Dr. Nida Shamim, FCPS. Department of Ophthalmology, Jinnah Post Graduate Medical Centre, Karachi, Pakistan; 2 Dr. Nausheen Hayat, FCPS. MRCSEd Opth. Department of Ophthalmology, Jinnah Post Graduate Medical Centre, Karachi, Pakistan; 3 Dr. Alyscia Cheema, FCPS, FRCS. Department of Ophthalmology, Jinnah Post Graduate Medical Centre, Karachi, Pakistan

**Keywords:** Congenital ptosis, Frontalis sling surgery, Visual acuity, Prolene

## Abstract

**Objective:**

To assess the visual outcome after sling procedure using prolene sutures in children with simple congenital ptosis.

**Methods:**

A descriptive case series study was performed in the Department of Ophthalmology of Jinnah Post Graduate Medical Centre, for a duration of six months in which 20 patients aged 3-10 years were selected with either unilateral or bilateral congenital ptosis. They were thoroughly examined and visual acuity and degree of ptosis were measured pre-operatively. Frontalis sling surgery was performed using prolene sutures on all patients after which their visual acuity and degree of ptosis were measured once again after three months post-operatively. Data were analyzed using SPSS version 20.0. For qualitative variables, frequency and percentages were calculated. Mean and Standard deviation was commutated for the quantitative variable.

**Results:**

The mean age of the patients was 8.15±1.75. 11 (55%) male and 9 (45%) female participants were included in the study. Visual acuity improved in all the patients with all the patients attaining a visual acuity of 6/6 (n=12, 60%), 6/9 (n=7, 35%), and 6/12 (n=1, 5%). The degree of ptosis post-operatively was not found in any patient either.

**Conclusion:**

Sling procedure helped in eliminating ptosis and improved visual acuity in patients with simple congenital ptosis.

## INTRODUCTION

The muscle of the upper eyelid levator palpebrae superioris is supplied by the oculomotor (CN III), keeping the upper eyelid elevated.^1^ However, sometimes the eyelid may drop leading to what is called Ptosis. Ptosis is the drooping or sagging of a body part.^2^ Ptosis is also known as blepharoptosis, is a condition in which there is an atypical low-lying upper eyelid margin in the primary gaze, ultimately leading to the narrowing of the palpebral opening and fissure. Ptosis is either presented from birth, known as congenital ptosis, or may go onto develop later in life. Congenital ptosis is a rare condition presenting with a loosening of the upper eyelids, impart due to the loss of muscular or nerve function that may be unilateral or bilateral.^3^,^4^ Improper data collection prevents us from detecting the proper incidence of congenital ptosis worldwide, with studies from various authors giving different incidence rates of congenital ptosis.^5^-^7^ Treatment of Ptosis is solely surgical; however, not all patients require surgical correction, although children with amblyopia may benefit from a surgical procedure. The procedure selected for surgical correction of ptosis solely depends on the degree of functionality of the levator muscle, along with the severity of ptosis. It is recommended that surgery should be deferred until the patient is 3-5 years of age. This helps to ensure facial growth and maturation and helps in attaining proper patient cooperation during preoperative evaluation.^8^ Commonly, Silicone and Prolene slings are used for children with congenital ptosis. This is because it doesn’t leave any scar and has been easily adjusted in the future.^9^ The Frontalis sling suspension is a customary indicated procedure in patients with congenital ptosis.

In Pakistan, congenital ptosis is prevalent, with a study showing it to be the third most common eye malformation in that particular study.^10^ Several materials are utilized in congenital ptosis treatment which includes autogenous materials such as fascia lata; Palmaris longus tendon and non-autogenous materials including nylon monofilament, mersilene mesh, silicone band, and more.^11^ However, in children there is not adequate Fascia lata that can be used in the sling procedure, therefore silicone and prolene slings may be appropriate for children as they don’t contribute to scar formation and can be later on adjusted.^12^ Once there is sufficient autogenous fascia available, they can be used for another sling procedure in the future. Considering the number of eye-related conditions prevalent in our country, we need to find the most efficient method, which can be affordable, convenient to treat congenital ptosis and prevents any future complications. Therefore, we decided to conduct a study in our department to assess the visual outcomes in children with congenital ptosis undergoing sling procedures with prolene sutures.

## METHODS

A descriptive case series study was conducted at the Department of Ophthalmology of Jinnah Post Graduate Medical Centre, for a duration of six months. The patients were selected based on a non-probability sampling technique in which 20 patients age 3-10 years suffering from either unilateral or bilateral simple congenital ptosis were selected and underwent frontalis sling surgery with prolene 3.0 sutures.

### Inclusion Criteria:

• Very weak levator function (<4 mm)
• Age of 3-10 years
• male or female
• Unilateral or bilateral
• Good bell’s phenomenon (more than +3) (4+ implies complete disappearance of the cornea and zero corresponds to absence of Bell’s phenomenon).

### Exclusion Criteria:

• Weak Bell’s phenomenon (<2)
• Lid disorders
• Jaw winking phenomenon (synkinetic movements of upper lid on masticating movements of the jaw)
• Impaired corneal sensitivity
• Systemic or myopathic disorders with secondary ptosis such as myasthenia gravis, myotonic dystrophy, chronic progressive external ophthalmoplegia (CPEO), Graves’ disease, assessed clinically.
• Any history of intra- or extraocular and eyelid surgery, and any history of sharp or blunt trauma to eyelids, eyelid tumors, and scars

### Ethical Considerations:

After proper approval was granted (Ref. No.F.2-81/2020-GENL/50742/JPMC, dated December 9, 2020) by the institutional review board of Jinnah Postgraduate Medical Centre, all the patients were referred for sling surgery. Written consent was acquired from all the participants in the study and parents of every patient were carefully briefed about the procedure and their involvement in the study.

### Assessment of patients were based on the following:

Performa’s were filled out, complete history was taken, Visual acuity was assessed by performing cycloplegic refraction and best-corrected visual acuity using the Snellen eye chart. All patients were examined for their anterior segment and posterior segment and collected over the basis of their levator function and degree of ptosis. Special ptosis examination included lid fissure height (normal 10mm), levator function (poor 0-3mm, fair 4-7mm, good 8-12mm and very good more than 13mm), upper lid margin-reflex distance (MRD1) normal 4mm, Bell’s phenomenon (4+ equals to complete disappearance and zero equals to no Bell’s phenomenon), and jaw winking. Severity of ptosis is graded on the basis of lid fissure height (the distance between the upper and lower lids), normally the upper lid rests 2mm below the upper limbus. Ptosis is mild upto 2mm, moderate 3mm and severe when more than 4mm. Severe ptosis of more than 5mm and poor levator function of less than 5mm are treated by frontalis sling procedure.

All patients were operated on for sling procedure by a single surgeon under general anesthesia. For the procedure, we used prolene suture 3.0 instead of fascia lata as there was insufficient tissue available from the subjects of this age group. Frontalis sling was performed using Fox Pentagon technique. The surgical procedure included two incisions at 5mm distance from the upper eyelid margin, and three incisions at the supraciliary border, two on the eyebrow laterally and one on the center of two. Pulling the prolene 3.0 suture through the incisions at suborbicularis plane will make a pentagon drawing the upper lid upwards adjusted to the required distance from the pupil. Finally securing the suture with the knots and burying in the skin. Patients were asked to follow-up at 1^st^ post-operative day, 1^st^ post-operative week, and monthly for three months.

During follow-up period the visual acuity was measured again by doing cycloplegic refraction and best corrected visual acuity by Snellen chart, amblyopia was diagnosed in few patients and were advised patching. The degree of ptosis was also assessed pre- and post-operatively. The compliance of patients was overcome by appropriate counselling. The data were analyzed using the Statistical Package of Social Science (SPSS) Version 20.0. For qualitative variables, frequency and percentages were calculated. Mean and Standard deviation was commutated for the quantitative variable.

## RESULTS

Twenty patients were included in this study, all of which had congenital ptosis unilaterally or bilaterally. Age of the patients included in this study ranged from three years to ten years (Average: 8.15±1.75). Eleven patients (55%) were female, while nine patients (45%) were male. Pre-operative Visual Outcome calculated through Snellen Eye Chart was 6/9 in four patients (20%), 6/12 in 3 patients (15%), 6/19 in 2 patients (10%), 6/24 in four patients (20%), 6/36 in three patients (15%), and 6/60 in four patients (20%). Post-operative Visual Outcome calculated through the Snellen eye chart was 6/6 in 12 patients (60%), 6/9 in seven patients (35%), 6/12 in one patient (5%). Pre-Operative degree of ptosis was calculated to be moderate in 12 patients (60%), and severe in 8 patients (40%). No degree of ptosis was seen in any of the patients post-operatively.

**Table-I:** Shows the frequency and percentage of age group among the patients.

Age (years)	Frequency	Percentage%
1-4	5	25
5-7	6	30
8-10	9	45

**Table-II:** Shows the frequency and percentage of pre- and post-operative (3^rd^ month) visual outcome.

Visual Acuity Snell’s Chart	Frequency and Percentage of Pre-operative visual outcome		Frequency and Percentage of Post-operative visual outcome	

	n	%	n	%
6/6	-	-	12	60
6/9	4	20	7	35
6/12	3	15	1	5
6/19	2	10	-	-
6/24	4	20	-	-
6/36	3	15	-	-
6/60	4	20	-	-

Total	20	100.0	20	100.0

**Fig.1:**
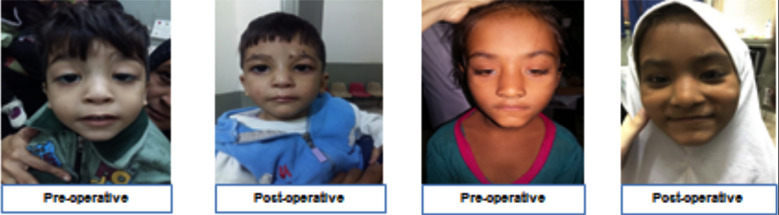
Shows pre and six weeks post-operative gross appearance of the patients.

## DISCUSSION

Congenital ptosis is present from the onset of birth; it is an inherited defect in the development of the levator muscle which can be unilateral or bilateral. If bilateral congenital ptosis is present the patient can choose to compensate for this anomaly by looking downward or elevating his chin, causing abnormalities in head and neck posture at whatever the age the condition appears along with a significant impact on the aesthetics of the patients.^13^ Early diagnosis and surgical treatment should be carried out as severe ptosis of the eye can lead to amblyopia which requires correction immediately to preserve vision.^14^

Our study had 12 (60%) patients with moderate, and 8 (40%) patients with severe ptosis among 11 (55%) male and 9 (45%) female patients. In Rodriguez et al. study’s, there were 63.6% patients with moderate ptosis and 36.4% patients with severe ptosis, the degree of ptosis is very similar to our study.^15^ We performed the sling procedure to correct congenital ptosis and assessed the degree of ptosis found after the correction of ptosis. The Frontalis sling procedure is strongly indicated in cases with poor or unapparent levator muscle function, as was in our study.

We saw a considerable amount of improvement in the visual acuity of the patients after the procedure, with all patients post-operatively achieving visual acuity of 6/6, 6/9, and 6/12. In Salcedo et al. study visual acuity improved in 100% of patients, to varying degrees. Before surgery, 72% had visual acuity of 0.1-0.5. Six months post-surgery, with visual rehabilitation, 90.9% exhibited visual acuity of >0.5. In 81.8% of patients, palpebral ptosis was fully corrected.^16^ Refractive defects can arise according to the severity of eyelid drooping, these refractive defects include astigmatism, strabismus, and the most serious of all amblyopia. Visual acuity improved significantly in our study, thereby demonstrating that deprivation was the cause behind the diminished vision, this is also apparent in the literature describing absent or inadequate stimulant as a direct factor for amblyopia.^17^-^19^

Locally studies have also been conducted to assess the functional results of sling operation. Waseem et al conducted a study on six severe cases of congenital ptosis that had levator palpebrae superioris function of 4mm or less, his study showed satisfactory results in terms of cosmetic and function.^20^ Moin et al. conducted a retrospective analysis to evaluate results of tarsal fixation in Frontalis sling ptosis surgery. The study concluded that there was reliable correction of poor function of ptosis.^21^ These studies coincide with our findings that this procedure is a reliable procedure, producing safe and effective results. Javaid et al studied the outcomes and complications of Frontalis suspension. He reached a conclusion that Frontalis suspension is an effective procedure for treating unilateral ptosis and neither is it associated with any serious complications.^22^ A similar study has been conducted locally that also used prolene suture 2/0 for unilateral ptosis with poor levator function, taking bigger age group range from two to 41 years with no idea how many children were included in the study and why fascia lata was not performed in ages more than 15 years.^23^

There are very less previous studies that have been conducted locally in Pakistan assessing the degree of ptosis and functional results of sling procedure with the prolene suture. Many of them have worked on levator resection for severe ptosis. Our study in particular focuses on the minor age group population that is three to 10 years that needs urgent surgical repair for better visual and cosmetic outcome taking unilateral or bilateral ptosis preventing the development of amblyopia. Furthermore, our study was specially designed precisely to generate results separately in both moderate and severe ptosis.

Prolene is a synthetic, non-absorbable monofilament suture used in surgical procedures. It is readily available, cost-effective, minimizes tissue reaction, and can easily be removed without any major scar formation. Non-autogenous materials are used for temporary measures until there is sufficient autogenous fascia available.^24^. The use of prolene sutures in Sling procedures for Congenital Ptosis is presumed to be a temporary method, however in our study it was highly favorable and produces optimistic results, and might give long-term results aswell.^25^

Our study demonstrated a reduction in ptosis, better cosmesis, as well as an improvement of visual outcome after sling surgery using prolene sutures. Future studies can be carried out to assess the complications and the effect of different materials that can be used in sling surgery.

### Limitations of the study:

It includes short follow-up as most of the patients were coming from rural areas of Sindh and other cities and small sample size.

## CONCLUSION

Improvement in visual acuity and complete removal of ptosis was seen after carrying out the sling procedure using prolene suture 3/0 in children with congenital ptosis. We obtained satisfactory results using prolene 3/0 suture in young children where facsia lata cannot be used. The suture is easily available and good eye symmetry is achieved. It can be used as a temporary treatment to prevent amblyopia in young years until the child is old enough to undergo permanent sling procedure where good amount of fascia lata can be readily harvested from the leg and patient will be fully aware to take appropriate postoperative care.

### Authors’ Contribution:

**NS** conceived, designed and did statistical analysis & editing of manuscript. She is also responsible for accuracy of study.

**NS and NH** did data collection and manuscript writing.

**AC** did review and final approval of manuscript.
